# Screening of a Focused Ubiquitin-Proteasome Pathway Inhibitor Library Identifies Small Molecules as Novel Modulators of Botulinum Neurotoxin Type A Toxicity

**DOI:** 10.3389/fphar.2021.763950

**Published:** 2021-09-27

**Authors:** Edanur Sen, Krishna P. Kota, Rekha G. Panchal, Sina Bavari, Erkan Kiris

**Affiliations:** ^1^ Department of Biological Sciences, Middle East Technical University, Ankara, Turkey; ^2^ Therapeutic Discovery Branch, United States Army Medical Research Institute of Infectious Diseases, Frederick, MD, United States; ^3^ Edge BioInnovation and Healion Bio, Frederick, MD, United States

**Keywords:** Botulinum Neurotoxin, Botulinum Neurotoxin Light Chain Degradation, Botulinum Inhibitors, celastrol, WP1130, PR-619, ubiquitin-proteasomal pathway

## Abstract

Botulinum neurotoxins (BoNTs) are known as the most potent bacterial toxins, which can cause potentially deadly disease botulism. BoNT Serotype A (BoNT/A) is the most studied serotype as it is responsible for most human botulism cases, and its formulations are extensively utilized in clinics for therapeutic and cosmetic applications. BoNT/A has the longest-lasting effect in neurons compared to other serotypes, and there has been high interest in understanding how BoNT/A manages to escape protein degradation machinery in neurons for months. Recent work demonstrated that an E3 ligase, HECTD2, leads to efficient ubiquitination of the BoNT/A Light Chain (A/LC); however, the dominant activity of a deubiquitinase (DUB), VCIP135, inhibits the degradation of the enzymatic component. Another DUB, USP9X, was also identified as a potential indirect contributor to A/LC degradation. In this study, we screened a focused ubiquitin-proteasome pathway inhibitor library, including VCIP135 and USP9X inhibitors, and identified ten potential lead compounds affecting BoNT/A mediated SNAP-25 cleavage in neurons in pre-intoxication conditions. We then tested the dose-dependent effects of the compounds and their potential toxic effects in cells. A subset of the lead compounds demonstrated efficacy on the stability and ubiquitination of A/LC in cells. Three of the compounds, WP1130 (degrasyn), PR-619, and Celastrol, further demonstrated efficacy against BoNT/A holotoxin in an *in vitro* post-intoxication model. Excitingly, PR-619 and WP1130 are known inhibitors of VCIP135 and USP9X, respectively. Modulation of BoNT turnover in cells by small molecules can potentially lead to the development of effective countermeasures against botulism.

## Introduction

Botulinum neurotoxins (BoNT) are the causative agents of botulism disease, which can be lethal mainly due to respiratory failure (Johnson, 2019). Their extreme toxicities and ease of production led to the classification of BoNTs as Category A bioterror agents, and BoNTs have been attempted for use as biological weapons in the past (Arnon et al., 2001). On the other hand, formulations of these toxins are extensively utilized in clinics as FDA-approved therapeutics to treat various conditions such as movement disorders as well as for cosmetic purposes (Choudhury et al., 2021). BoNT intoxication primarily leads to inhibition of the motor neuron-muscle connectivity by cleaving specific SNARE proteins crucial for neuroexocytosis (Montal, 2010). After these toxins have gained entry into motor neurons, there are no treatment options to inhibit their enzymatic activity inside the cell (Lin et al., 2019). There are at least seven different BoNT serotypes with an increasing number of subtypes and chimeric toxins (Dong and Stenmark, 2021), of which serotypes A, B, E, and F lead to human botulism (Maslanka, 2014). Among these, serotype A (BoNT/A) causes the most human botulism cases, and it is the most utilized BoNT serotype in clinical formulations (Choudhury et al., 2021). This serotype has the most prolonged half-life in neurons compared to other serotypes (Shoemaker and Oyler, 2013). For example, both BoNT/A and another human botulism-causing serotype, BoNT/E, target the same protein, SNAP-25, in neurons; however, BoNT/E is destroyed naturally within a few days to weeks while BoNT/A can be active for up to 6 months in the neuronal cytosol (Shoemaker and Oyler, 2013).

Recent work elucidated the molecular mechanisms by which BoNT/A Light Chain (A/LC) evades the ubiquitin-proteasome pathway (UPP) in neurons (Tsai et al., 2017). More specifically, A/LC is ubiquitinated by the E3 ligase, HECTD2, but it is protected from proteasomal destruction by the dominant effect of VCIP135/VCPIP1, a deubiquitinase (DUB) (Tsai et al., 2017). Notably, previous work provided proof-of-concept that A/LC turnover in cells can be modified by increasing its ubiquitination via designer E3 ligases (Tsai et al., 2010) or by delivering fusion proteins, including a single-chain antibody specific to A/LC fused to an F-box domain recognized by an endogenous E3-ligase (Kuo et al., 2011). However, to pave the road for drug development studies, it is crucial to identify small molecules that can modulate UPP to affect A/LC toxicity. There have been extensive drug development efforts against BoNT/A, and although elegant work from many groups has identified various compound classes as BoNT/A inhibitors, none has graduated to clinical trials yet (Lin et al., 2019). To the best of our knowledge, targeting UPP with small molecules to modulate the persistence of A/LC has not been explored. Although UPP is considered a highly challenging drug target, there are already FDA-approved and many promising clinical/preclinical stage compounds targeting different components of UPP (Wu et al., 2020). For example, proteasome inhibitors Bortezomib, Carfilzomib, Ixazomib, and E3 modulators Thalidomide, Lenalidomide, and Pomalidomide are FDA approved (Wu et al., 2020). In the UPP pathway, DUBs are considered attractive drug targets with significant clinical potential as it may be possible to develop selective DUB modulators due to their diversity (about 100 DUBs in humans) (Basar et al., 2021), and well-defined catalytic clefts (Clague et al., 2019). Indeed, selective DUB modulators, although a few, have been developed (Harrigan et al., 2018; Clancy et al., 2021).

In this work, we focused on identifying small molecules to potentially affect ubiquitination and/or deubiquitination of the A/LC to modulate the A/LC activity in cells. Previous work demonstrated that, in addition to VCIP135/VCPIP1, another DUB, USP9X, potentially indirectly affects the stability of A/LC in cells (Tsai et al., 2017), suggesting that there may be other crucial, indirect players in this process. Therefore, we sought to utilize a more general approach rather than focusing on just the known UPP factors for A/LC turnover and screened a focused UPP inhibitor library including VCIP135 and USP9X inhibitors to identify compounds modulating BoNT/A toxicity in cells. Our initial screen utilizing mouse embryonic stem cell-derived motor neurons identified ten potential lead compounds affecting BoNT/A holotoxin mediated SNAP-25 cleavage in neurons in a pre-intoxication experimental model. Then, we examined the dose-dependent effects of the selected compounds and tested their effects on cell viability. We also explored the effects of the lead compounds on the stability and the ubiquitination of the A/LC in cells. Among the identified small molecules, PR-619 and WP1130 (degrasyn) appear to be highly crucial as they are the known inhibitors of VCIP135 and USP9X, respectively, which have been identified as DUBs affecting the half-life of A/LC (Tsai et al., 2017). Importantly, PR-619, WP1130, and Celastrol also exhibited efficacy against BoNT/A holotoxin in post-intoxication experimental conditions.

## Materials and Methods

### Compound Library

**FIGURE 1:**
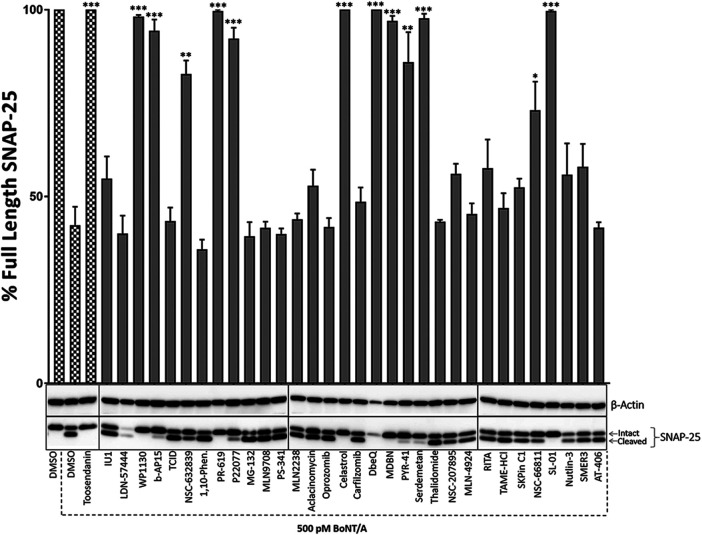
The initial screen of the small molecule UPP inhibitor library for BoNT inhibition in mouse ES-derived motor neurons in pre-intoxication conditions. Mouse ES-derived motor neurons were treated with small molecules (20 µM) for 30 min and then intoxicated with BoNT/A holotoxin (500 pM) for 4 h. Western blotting was utilized to calculate percent full-length (intact) SNAP-25 cleavage in each condition. Toosendanin, a known BoNT inhibitor, was used as a positive control. β-Actin was used as a loading control. Data are presented as means ± SEM calculated from three independent experiments and compared by Student’s *t*-test to DMSO+500pM BoNT/A condition. ★★★, ★★, ★ Value significant at 99.9%, 99%, and 95 %confidence level, respectively, compared to DMSO + Toxin control conditions.

The ubiquitin-proteasome pathway inhibitor library was purchased from LifeSensors (#SI9032), which contained 32 UPP inhibitors. The library comprises small molecules targeting many components in UPP (i.e., E1, E3, proteasome), including DUB inhibitors. Compound stock concentrations in the library were 10 mM, dissolved in dimethyl sulfoxide (DMSO). All of the compounds in the purchased library were tested in Figure 1.

### Differentiation of Mouse Embryonic Stem Cells Into Motor Neurons

Directed differentiation of mouse ES (HBG3) cells towards motor neurons were based on our previously established and characterized protocols (Kiris et al., 2011; Kota et al., 2014). Briefly, HBG3 ES cells were co-cultured with mitomycin-inactivated mouse embryonic fibroblast (mitoMEF), using mouse ES medium consisting of DMEM supplemented with 15% fetal bovine serum (FBS), 1,000 Unit/ml Leukemia Inhibitory Factor (LIF), β-mercaptoethanol (final concentration 0.1 mM), 1% Penicillin-Streptomycin, 1% Glutamax, and 1% Non-essential amino acids. For motor neuron differentiation, the mouse ES cells were separated from mitoMEFs via enzymatic methods, and embryoid bodies (EBs) were formed (Day 0) using the differentiation medium, which was composed of 1:1 Advanced DMEM-F12 and Neurobasal medium supplemented with 1% Penicillin-Streptomycin, 1% Glutamax, β-mercaptoethanol (final concentration 0.1 mM), and 10% Knockout Serum Replacement, using low-attachment dishes. On differentiation day 1, the EBs were collected and cultured in a fresh differentiation medium for 24 h. For the induction of neuralization, the EBs were then treated (Day 2) with Retinoic Acid (1 μM, Sigma) for 24 h. On days 3 and 4, Hh-Ag1.5 (Cellagentech) (1 µM final concentration) was utilized to induce motor neuron specification. On day 5, EBs were transferred to new plates with fresh differentiation medium, supplemented with 2% B-27 serum-free supplement (Invitrogen), brain-derived neurotrophic factor (10 ng/ml, Chemicon), glial cell-derived neurotrophic factor (100 ng/ml, R&D Systems), ciliary-derived neurotrophic factors (10 ng/ml, Chemicon), and Neurotrophin3 (10 ng/ml, Chemicon). On day 7, EBs were dissociated using accutase, counted, and plated to matrigel (BD Biosciences) coated dishes for 3 days of neurite elongation. Unless otherwise stated, all reagents were purchased from Thermo Fisher Scientific.

### BoNT/A Intoxication, Compound Treatments, and Western Blotting

For BoNT/A holotoxin (Metabiologics) experiments with pre-intoxication conditions (Figures 1, 3), mouse ES cell-derived motor neurons were cultured in 24-well plate formats and pre-treated with compounds and cultured for 30 min at 37°C with 5% CO_2_ prior to intoxication_,_ similar to previous studies (Kiris et al., 2015a; Kiris et al., 2015b). Neurons were then intoxicated with the holotoxin for 4 h in a humidified incubator with 5% CO_2_ at 37°C. For the post intoxication conditions in Figure 7; neurons were first intoxicated with indicated amounts of BoNT/A holotoxin, cultured for 30 min, and then the compounds were supplied to the intoxicated cultures. Total intoxication time was kept as 4 h. At the end of the holotoxin experiments in both pre- and post-intoxication conditions, the toxin was removed through approved protocols, cell lysates were prepared, and the total protein content was evaluated by Bradford assays. The degree of SNAP-25 cleavage was determined using western blotting with SNAP-25 antibodies (SMI-81, BioLegend). Briefly, samples were subjected to the electrophoresis using 12% Tris-Glycine gels, and the transfer for western blotting was conducted in wet conditions using PVDF membranes at 30 V for 2 h, using standard protocols. The membranes were blocked in 5% skimmed milk dissolved in 0.01% TBS-T and then incubated in the primary antibody solution overnight at 4°C. The membrane was washed two times, re-blocked, and then incubated in secondary antibody solution for 1 h at room temperature. The enhanced chemiluminescence (ECL) substrate was used for visualization, and the imaging and analyses were carried out using a SynGene GeneGnome Chemiluminescence Imaging System.

### MTT Assay

The human embryonic kidney cell line (HEK293) was cultured in a growth medium consists of DMEM supplemented with 10% Fetal Bovine Serum, 1% Glutamax, and 1% Penicillin-Streptomycin, at 37°C with 5% CO_2_. HEK293 cells were plated in 96-well culture plates and incubated for 24 h at 37°C, 5% CO_2_. The cells were treated with the compounds at increasing concentrations (1.25, 2.5, 5, 10, and 20 µM), and incubated for 4 h. Then, 3-(4,5-Dimethylthiazol-2-yl)−2,5 diphenyltetrazolium bromide (MTT) solution was added to the cells, followed by the addition of 1% SDS-0.01M HCl and the cells were incubated for 18 h. The absorbance measurement was conducted using a microplate reader (Thermo Fisher Scientific) at 570 nm. The readings were normalized to the blank controls and compared to control conditions to calculate percent cell viability for each condition.

### Cycloheximide Chase Experiments

HEK293 cells were seeded in 24-well plates for 24 h and then transfected with 1 µg plasmid encoding YFP-tagged A/LC (a kind gift from Dr. Yien Che Tsai, NCI-Frederick, Frederick, MD, United States), using TurboFect Transfection Reagent according to the manufacturer’s instructions. Cycloheximide (CHX) (25 μg/ml) (Sigma-Aldrich, C7698) was added to the transfected cells to inhibit new protein synthesis 24 h post-transfection. The time point that CHX added was collected as “CHX 0 h control”. Cells were then incubated in CHX for 2 h in a humidified incubator with 5% CO_2_ at 37°C. After 2 h, the media containing the CHX was removed, and the indicated compounds (20 µM) were applied to the cultures, which is designated as “Compound Addition 0 h” time point. The cells were further incubated and lysed at the specified time points indicated in Figure 5 in NP-40 lysis buffer (5 M NaCl, 10% NP-40, 1 M Tris pH 8.0), containing freshly added protease and phosphatase inhibitors. Western blotting was utilized using GFP antibodies (Invitrogen, A11122 and Santa Cruz Biotechnologies, sc-9996) to determine A/LC degradation. Each western blot was stripped and reprobed with β-Actin as the loading control, and the data quantification (Figure 5B) was conducted by normalizing the GFP levels to β-Actin of each individual blot.

### Ubiquitination Assay

To determine the ubiquitination of BoNT/A LC, HEK293 cells were co-transfected with HA-tagged Ubiquitin (a kind gift from Dr. Lino Tessarollo, NCI-Frederick, Frederick, MD, United States) and YFP-tagged A/LC using TurboFect Transfection Reagent and cultured for 40 h. The cells were treated with the compounds (20 µM) and harvested after 30 min and 3 h post-treatment, using ubiquitination lysis buffer (30 mM Tris. HCl pH8, 75 mM NaCl, 10% Glycerol, 1% Triton X-100, and freshly added protease inhibitor and phosphatase inhibitor). Lysates were then processed and precleared, followed by YFP-LCA immunoprecipitation with a monoclonal GFP antibody (Santa Cruz Biotechnologies, sc-9996) or a control IgG (normal mouse IgG, Santa Cruz Biotechnologies, sc-2025), using protein G magnetic beads (SureBeads) (BioRad), according to the manufacturer’s IP protocol. Following, samples were subjected to western blotting, and ubiquitination levels were determined using HA antibodies (Sigma, H6908), similar to previous studies (Tsai et al., 2010; Tsai et al., 2017). Each western blot was stripped and re-probed with GFP antibodies. The data were quantified by normalizing the ubiquitination signal to GFP levels on the same blot.

## Results

### Screening of a Small Molecule UPP Inhibitor Library for BoNT Holotoxin Inhibition in Mouse ES-Derived Motor Neurons at Pre-intoxication Conditions

**FIGURE 2:**
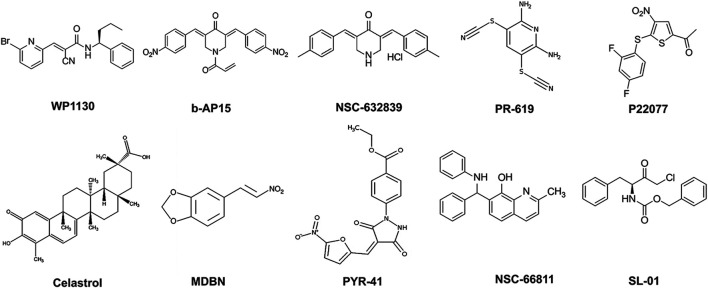
2D-Chemical structures of the lead compounds. WP-1130, b-AP15, NSC 632839, PR-619, P22077, Celastrol, MDBN, PYR-41, NSC 66811, and SL-01.

To test the potential inhibitory effects of the small molecules targeting UPP against BoNT/A, we examined the effects of each compound in the library on BoNT/A mediated cleavage of SNAP-25 in mouse ES-derived motor neurons. It is well established that SNAP-25 is the only known biological target of BoNT/A, and the toxin-mediated SNAP-25 protein cleavage is routinely used as a read-out to measure the toxin’s biological activity in cells (Kiris et al., 2014a). BoNT/A removes nine amino acids from the C-terminal end of SNAP-25, and the resulting large fragment can be separated from intact SNAP-25 via SDS-PAGE, and the percentage of full-length (uncleaved) SNAP-25 is typically calculated via western blotting as a measurement of toxin activity (Kiris et al., 2011). Given that BoNT/A naturally targets motor neurons, mouse ES-derived motor neurons serve as physiologically relevant mammalian cell culture systems (Kiris et al., 2014a). We conducted the initial screen at pre-intoxication conditions in which mouse ES-derived motor neurons were cultured in 24-well plates and treated with small molecules (20 µM) for 30 min, and then the cells were intoxicated with BoNT/A holotoxin (500 pM) for 4 hours. Our western blot analyses demonstrated that WP1130, b-AP15, NSC-632839, PR-619, P22077, Celastrol, MDBN, PYR-41, Serdemetan, NSC-66811, and SL-01 statistically significantly protect SNAP-25 against BoNT/A holotoxin (Figure 1). Since two small molecules (LDN-57444 and DbeQ) affect the SNAP-25 and/or β-actin protein levels, they were excluded from further analyses. Neurons were morphologically examined in each experimental condition with compounds, and Serdemetan was removed from the pool of selected compounds because of its potential toxic effects on neurons based on morphological analyses and additional tests exhibiting inconsistent results. Toosendanin treatment (1 µM) was used as a positive control group (Shi and Wang, 2004), and as expected, it demonstrated complete protection against the toxin. The most exciting part of these results is that PR-619, known to affect VCIP135 (Tsai et al., 2017), inhibited BoNT/A holotoxin mediated SNAP-25 cleavage (Figure 1). Another small molecule, WP-1130, which affects USP9X, also exhibited inhibitory activity against BoNT/A in neurons. Taken together, WP1130, b-AP15, NSC632839, PR-619, P22077, Celastrol, MDBN, PYR-41, NSC66811, and SL-01, were selected for further studies based on our initial screen. 2D-structures of the lead compounds are given in Figure 2.

### Selected Compounds Exhibit Dose-dependent Effects Against BoNT/A Holotoxin in Neurons

**FIGURE 3:**
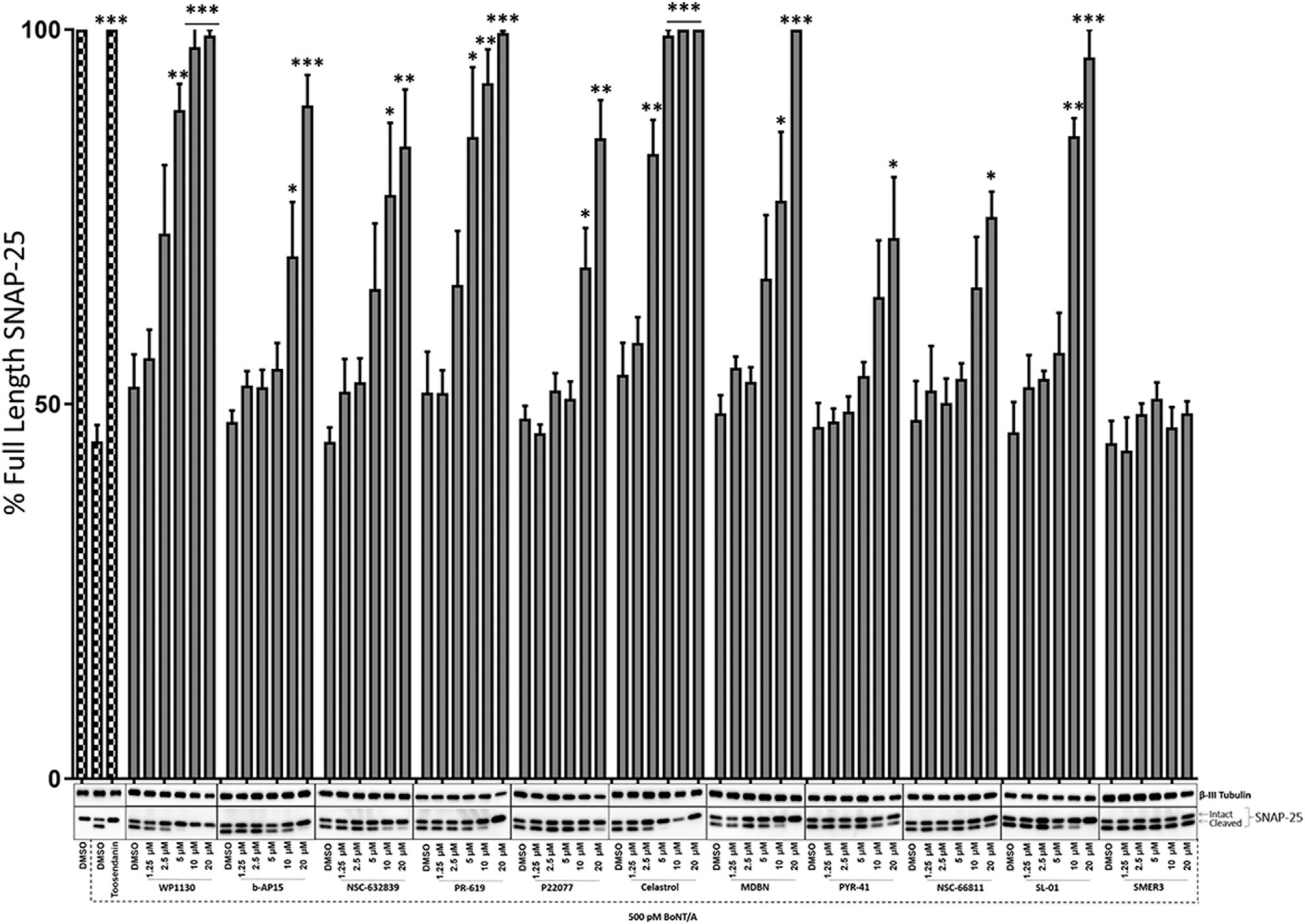
Dose-dependent effects of the selected compounds against BoNT/A holotoxin mediated SNAP-25 cleavage. Mouse ES-cell-derived motor neurons were treated with the compounds at increasing doses (1.25, 2.5, 5, 10, and 20 µM) for 30 min before intoxication with 500 pM BoNT/A holotoxin. The total intoxication time was 4 h. Toosendanin treatment (1 µM) was utilized as a positive control. β-III Tubulin was used as a loading control. Western blots are representatives of three independent experiments, and the data are presented as means ± SEM. ★★★, ★★, ★ Value significant at 99.9%, 99%, and 95 %confidence level, respectively, compared to DMSO + Toxin control condition of each compound set.

Selected compounds were then evaluated for their potential dose-dependent effects on the inhibition of BoNT/A mediated SNAP-25 cleavage in pre-intoxication conditions, similar to Figure 1. The ES-cell-derived motor neurons were treated with the compounds at varying concentrations (1.25, 2.5, 5, 10, and 20 µM) 30 min before 500 pM BoNT/A holotoxin intoxication of 4 hours. Similar to the initial screen, SNAP-25 cleavage was utilized as a read-out, and the effectiveness of the compounds was tested by determining the percentage of full-length SNAP-25 in each experimental condition compared to DMSO control conditions run with each compound set (Figure 3). SMER3, which did not show significant protection in the initial screen (Figure 1), was utilized as a negative control, which, as expected, did not lead to dose-dependent protection against BoNT/A holotoxin (Figure 3). Toosendanin treatment (1 µM) was used as a positive control, which exhibited complete protection. Excitingly, some of our lead compounds led to dose-dependent protection against the BoNT/A challenge, and 20 µM was the most effective dose for the most efficacious compounds to inhibit BoNT/A mediated SNAP-25 cleavage (Figure 3). In particular, Celastrol exhibited statistically significant protection at lower µM (as low as 2.5 µM) concentrations. Similarly, WP1130 and PR-619 affected BoNT/A mediated cleavage of SNAP-25 in a dose-dependent manner, and the results were statistically significant at as low as 5 μM b-AP15, NSC-632839, P22077, MDBN, and SL-01 also inhibited BoNT/A mediated SNAP-25 proteolysis in a dose-dependent manner but to a lesser extent. Inhibitory effects of PYR-41 and NSC66811 were only significant at 20 µM concentrations (Figure 3). Taken together, these analyses demonstrated that some of the lead compounds, including Celastrol, WP1130, and PR-619, are efficacious against BoNT/A holotoxin in a dose-dependent manner in pre-intoxication conditions.

### The Effects of Selected Compounds on the Cell Viability

**FIGURE 4:**
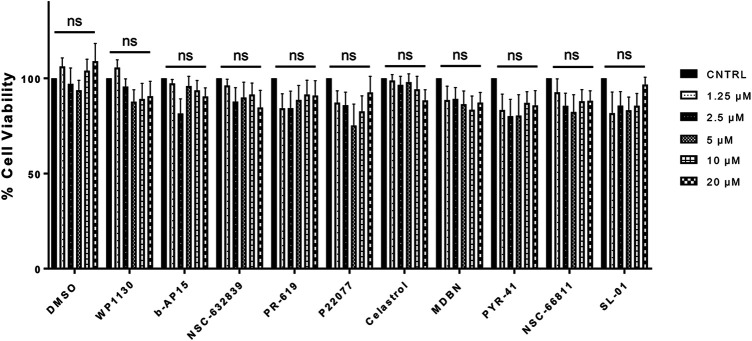
The Effects of Selected Compounds on the Cell Viability. MTT assay was performed using HEK293 cells treated with selected compounds at 1.25, 2.5, 5, 10, and 20 µM concentrations. Data are presented as means ± SEM calculated from four independent experiments and compared by Student’s *t*-test, using GraphPad Prism. “ns” stands for not significant.

We performed an MTT assay using HEK293 cells to determine whether the compounds have any potential toxic effects on cells. HEK293 cells are commonly utilized in viability measurements upon compound treatments (Class et al., 2015). HEK293 cells were treated with selected compounds at 1.25, 2.5, 5, 10, and 20 µM concentrations for 4 h, followed by MTT treatment and incubation for 18 additional hours. The control group was treated with DMSO as the compounds were dissolved in DMSO. We have not detected statistically significant viability differences in any of the compound-treated conditions compared to the control conditions (Figure 4).

### Selected Compounds Enhances the Degradation of BoNT/A LC in Cells

**FIGURE 5:**
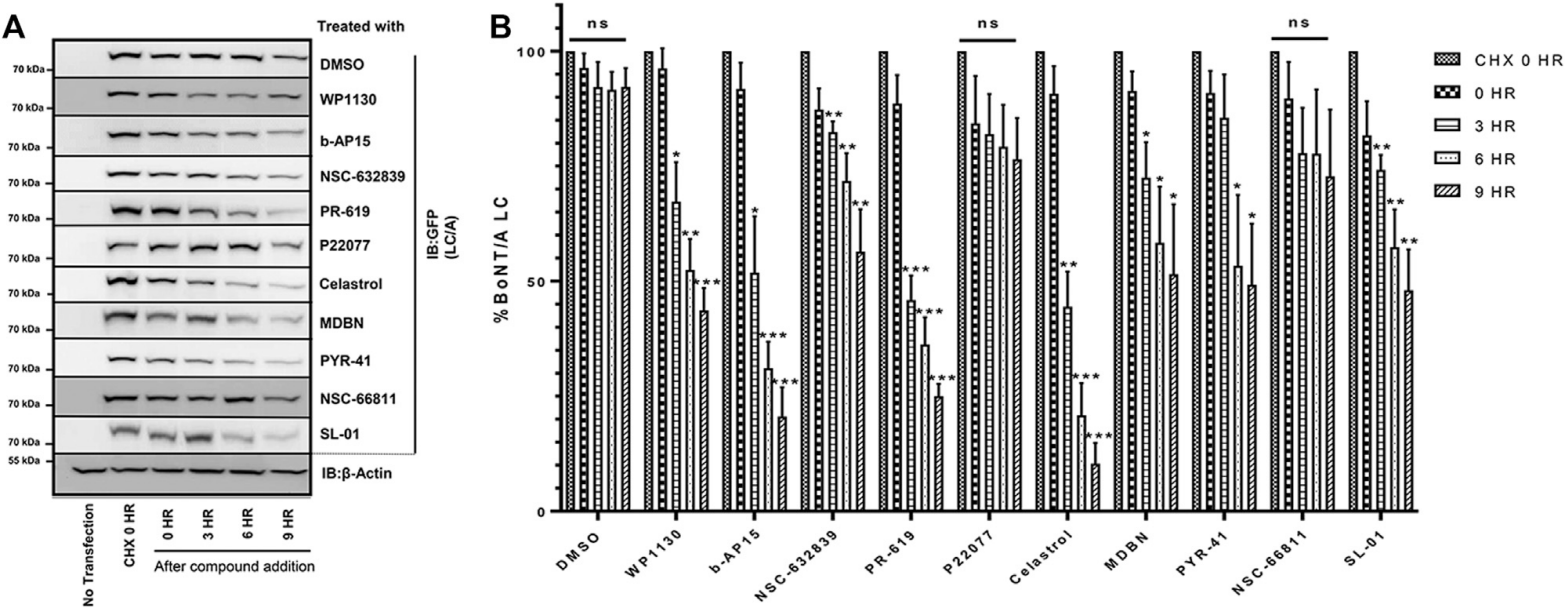
Selected Compounds Enhance the Degradation of BoNT/A LC in Cells **(A)** Representative western blot images demonstrating YFP-LCA protein levels at the indicated time points. HEK293 cells were transfected with YFP-LCA and after 24 h incubated with CHX. After 2 h of incubation with CHX, media was removed, and selected compounds (20 µM) were added to the cells and incubated for the indicated time points. “CHX 0 h” indicates the time point when CHX was added. “Compound treatment 0 h” indicates the time point when compound treatment was conducted. YFP-LCA expression levels were detected using an anti-GFP antibody. Each western blot was stripped and reprobed with β-Actin as the loading control, and the figure includes a representative β-Actin. **(B)** Quantitative analysis of YFP-LCA protein levels for each compound at the indicated time points. The data quantification was conducted by normalizing the GFP levels to β-Actin of each individual blot. Data are presented as means ± SEM calculated from three independent experiments and compared to corresponding “CHX 0 h” control conditions of each set, using GraphPad Prism (Student’s *t*-test). ★★★, ★★, ★ Value significant at 99.9, 99, and 95% confidence level, respectively. “ns” stands for not significant.

To investigate the effects of selected compounds on the degradation of the catalytic domain of BoNT/A, we sought to measure the changes in the protein level of YFP tagged BoNT/A LC at various time points after compound treatments. To do so, we utilized cycloheximide (CHX), a protein synthesis inhibitor, and performed chase experiments similar to previous studies (Tsai et al., 2017). “CHX 0 h” indicates the time point when CHX was added to HEK293 cultures after 24 h of post-transfection of YFP tagged BoNT/A LC. After 2 h of CHX treatment, cells were washed thoroughly, and selected compounds (20 µM) were administered to the cultures. Then, the samples were collected at various time points (0, 3, 6, and 9 h) to determine the effects of compounds on BoNT/A LC protein levels in cells. Cycloheximide is typically utilized at 50 μg/ml concentrations (Tsai et al., 2017; Tsai et al., 2010); however, we utilized a lower concentration (25 μg/ml) to achieve a relatively less A/LC degradation for easier detection of the effects of the compounds. Figure 5A shows representative YFP-LCA protein levels at indicated time points detected by western blotting using an anti-GFP antibody for each selected compound. DMSO treatment was utilized as a negative control. Our data demonstrate that eight out of ten tested compounds led to significant and time-dependent degradation of BoNT/A LC in cells (Figures 5A,B). For example, PR-619, WP1130, b-AP15, and Celastrol exhibited a highly significant effect on the degradation of A/LC, especially at 6 and 9 h time points. This finding is exciting as PR-619 and WP1130 inhibit VCIP135 and USP9X, respectively, which regulate the half-life of A/LC (Tsai et al., 2017), as mentioned above. NSC-632839, MBDN, PYR-41, and SL-01 also demonstrated efficacy on A/LC degradation but to a lesser extent. Interestingly, two compounds, P22077 and NSC66811, did not result in significant degradation of BoNT/A LC, although they inhibited the toxin in holotoxin experiments, suggesting that they may lead to inhibition through other unknown mechanisms than directly affecting the ubiquitination of the A/LC. Taken together, some of the lead compounds exhibited a highly significant effect on A/LC degradation in cells.

### Selected Compounds Promote the Ubiquitination of BoNT/A LC in Cells

**FIGURE 6:**
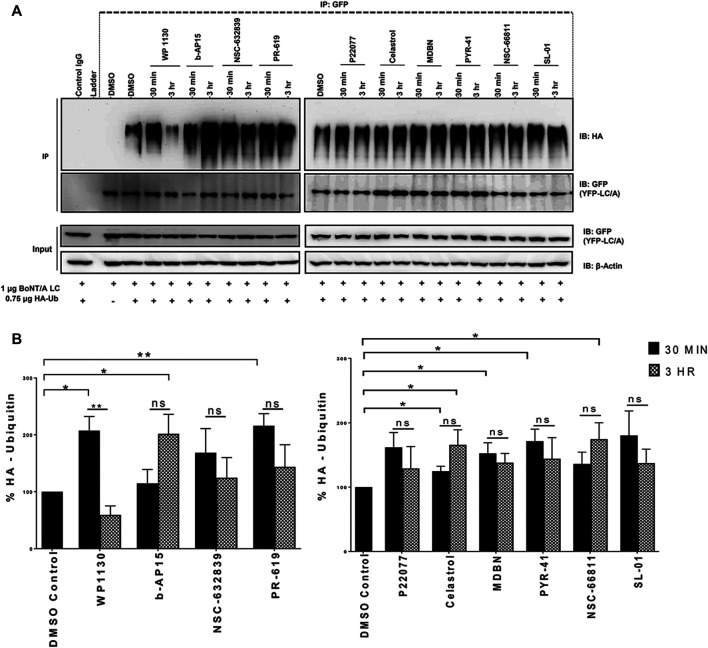
Selected Compounds Promote the Ubiquitination of BoNT/A LC in Cells. **(A)** HEK293 cells were co-transfected with HA-Ubiquitin and YFP-tagged BoNT/A LC plasmids for 24 h, then treated with the compounds (20 µM), and samples were collected at the indicated time points. YFP-tagged BoNT/A LC was immunoprecipitated with GFP antibodies or a normal mouse control IgG, and an HA antibody was utilized to detect ubiquitination. Each blot was stripped and re-probed with GFP antibodies. **(B)** Quantitative analysis of YFP-LCA ubiquitination levels in each compound treated conditions as compared DMSO control condition at the indicated time points. Data in each blot was normalized to corresponding GFP (YFP-A/LC) levels. Data are presented as means ± SEM calculated from three independent experiments and compared by Student’s *t*-test, using GraphPad Prism. “ns” stands for not significant.

We then sought to determine the effects of selected compounds on the BoNT/A LC ubiquitination in cells. BoNT/A LC ubiquitination experiments were conducted similar to previous studies (Tsai et al., 2010; Tsai et al., 2017). HEK293 cells transfected with HA-Ubiquitin and YFP-tagged BoNT/A LC plasmids were treated with the compounds (20 µM) at indicated time points. We have not utilized CHX to inhibit new protein synthesis in these experiments, so the cells were only treated with the compounds in question. BoNT/A LC was immunoprecipitated with GFP antibodies, and samples were subjected to western blotting with HA antibodies to detect ubiquitination, similar to previous studies (Tsai et al., 2010; Tsai et al., 2017). Each individual western blot was stripped and reprobed with GFP antibodies, and the data were quantified by normalizing the ubiquitination signal to GFP levels on the same blot. The DUB inhibitors WP1130 and PR-619 statistically significantly enhanced A/LC ubiquitination in 30 min compared to DMSO control conditions (Figures 6A,B), and the ubiquitinated A/LC levels were decreased at 3 h time points compared to 30 min time points, which may suggest degradation of A/LC as observed in Figure 5, although the decrease for PR-619 was not statistically significant (Figure 6B). Celastrol led to statistically significant A/LC ubiquitination at 30 min time point and a trend of gradual accumulation at 3 h time point compared to 30 min. Similarly, b-AP15 and NSC-66811 treatments resulted in enhanced and accumulated A/LC ubiquitination, which was statistically significant at 3 h time points for both. MDBN and PYR-41 also enhanced the ubiquitination of the A/LC, which was statistically significant for both at 30 min time points, while NSC-632839, P22077, and SL-01 did not exhibit a statistically significant effect (Figures 6A,B).

### WP1130, PR-619 and Celastrol Are Effective Against BoNT/A Post-intoxication

**FIGURE 7:**
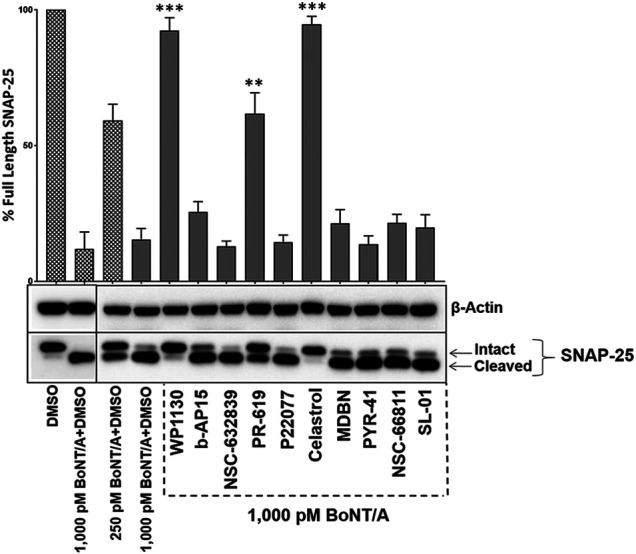
WP1130, PR-619, and Celastrol are effective against BoNT/A post-intoxication. Mouse ES cell-derived motor neurons were exposed to BoNT/A holotoxin at indicated concentrations for 30 min and then treated with 20 µM of the selected UPP modulators. After 4 h of total intoxication, cell lysates were prepared and subjected to western blotting to determine the degree of SNAP-25 cleavage. The blots were stripped and re-probed with β-Actin antibodies as the loading control. The values are given as mean ± SEM from three independent experiments. Statistical significance was calculated for each compound treatment as compared to DMSO + toxin only (1,000pM BoNT/A) control condition run on the same blot, using GraphPad Prism (Student’s *t*-test). ★★★, ★★ Value significant at 99.9 and 99% confidence level, respectively.

BoNT/A is well known for its long-lasting effects in the cytosol, and it is crucial to identify compounds that can inhibit BoNT/A enzymatic activity in intoxicated neurons. To determine whether selected UPP modulators can inhibit BoNT/A mediated SNAP-25 cleavage in post-intoxication conditions, we first intoxicated mouse ES-derived motor neurons with the indicated amounts of BoNT/A holotoxin and then treated the cultures with the compounds (20 µM) for 30 min after the intoxication started. The compounds were tested against a higher BoNT/A holotoxin challenge (1,000 pM) as opposed to 500 pM concentrations utilized in Figures 1, 3. Our results demonstrated that three compounds, WP1130, PR-619, and Celastrol, statistically significantly inhibited BoNT/A mediated SNAP-25 cleavage post-intoxication (Figure 7).

## Discussion

This work identified small molecule UPP inhibitors as novel modulators of BoNT/A in cells. There is an urgent need for therapeutic modalities to inhibit BoNT/A intoxication, especially after the toxin enters the neurons, where it can exhibit its toxic activity for up to 6 months (Lin et al., 2019). This is important because BoNT/A formulations are extensively used in clinics (Choudhury et al., 2021), and the wide distribution leads to concerns regarding accidents. Also, BoNT/A intoxications can naturally occur through food or liquid contaminations (Thirunavukkarasu et al., 2018). In addition, there is a potential for misuse of this relatively easy-to-produce toxin (Arnon et al., 2001). Various classes of small molecules inhibiting the toxin directly or through neuronal processes that may be curial for intoxication and/or recovery have been identified (Videnovic et al., 2014; Azarnia Tehran et al., 2015; Seki et al., 2015; Bremer et al., 2017a; Bremer et al., 2017b; Konstantinovic et al., 2018; Lin et al., 2019; Vazquez-Cintron et al., 2020; Patel et al., 2021). However, none of those candidates have yet progressed to FDA-approved drugs (Lin et al., 2019), suggesting the need for novel approaches.

One potential approach to modulate BoNT/A intoxication in cells would be targeting the persistence of A/LC (Kiris et al., 2014b). Previous work employing designer E3 ligases or A/LC specific single-chain antibodies fused to an F-box domain demonstrated that it is possible to expedite A/LC degradation in cells (Tsai et al., 2010; Kuo et al., 2011). Foreign proteins in normal functioning cells can be labeled with ubiquitin molecules for degradation by the UPP (Landré et al., 2014). However, BoNT/A can remarkably escape from this system (Tsai et al., 2017). Serotype A is the longest-lasting serotype in human botulism; it can survive within the neurons for up to 6 months (Shoemaker and Oyler, 2013). However, serotype E is active for just a few days to weeks, although it targets the same protein, SNAP-25, as BoNT/A (Shoemaker and Oyler, 2013). A recent study shows that the E3 ubiquitin ligase TRAF2 mediated ubiquitination destroys BoNT/E in neurons (Tsai et al., 2010). BoNT/A is also efficiently ubiquitinated by the E3 ligase HECTD2; however, the deubiquitinase VCIP135 removes the ubiquitin and thereby stabilizes the toxin by preventing its degradation (Tsai et al., 2017). Therefore, HECTD2 and VCIP135 activities appear to be essential for determining the lifetime of BoNT/A and the duration of its action in neurons. It is also important to mention that VCIP135 may not be the only DUB affecting A/LC half-life as it is shown that there is another DUB, USP9X, which might have an indirect effect on BoNT/A LC degradation (Tsai et al., 2017). The half-life of BoNT/A in cells can be potentially controlled if the E3 ligase and/or the DUBs can be manipulated.

Ubiquitination is a crucial post-translational process controlling many functions in cells, including protein turnover, localization, and endocytosis (Oh et al., 2018). Importantly, this is a reversible process, and DUBs can remove ubiquitin chains from the substrates (Clague et al., 2019). Many components in the UPP pathway have been targeted for drug development for various diseases, including cancer and neurodegenerative conditions. Such efforts led to the development of FDA-approved UPP targeting therapeutics. For example, proteasome inhibitors Bortezomib, Carfilzomib, Ixazomib, and E3 modulators Thalidomide, Lenalidomide, and Pomalidomide are FDA approved while there are many UPP targeting molecules, including DUB modulators, that are either in clinical trials and or in preclinical stages (Wu et al., 2020). Although proteasome and E1 targeting small molecules may lead to cellular toxicity due to non-specific effects, recent work has focused on developing specific E3 ligase and DUB modulators, and it has been shown that selective DUB inhibitor development is possible (Harrigan et al., 2018). DUBs are attractive drug targets due to their diversity and well-defined catalytic clefts (Clague et al., 2019; Basar et al., 2021), and indeed selective deubiquitinase inhibitors have been identified (Clancy et al., 2021; Varca et al., 2021).

Among the identified BoNT inhibitors in this study, PR-619 and WP1130 are of high interest as these are both DUB inhibitors with targets relevant to BoNT/A LC modulation. PR-619 is known to inhibit VCIP135 (Altun et al., 2011), which is previously identified as the main DUB inhibiting the degradation of BoNT/A LC (Tsai et al., 2017). PR-619 is a cell-permeable and reversible DUB inhibitor (Altun et al., 2011; Tian et al., 2011). It has been shown that PR-619 can enhance the ubiquitination of specific proteins, such as Bcl-2, and thereby enhance its degradation (Kuo et al., 2019), which is consistent with our findings (Figures 5, 6). WP1130, also known as degrasyn, is also a cell-permeable, reversible, small molecule that is considered the best described USP9X inhibitor (Harrigan et al., 2018). It has been shown that WP1130 can lead to enhanced ubiquitination and proteasome-dependent degradation of USP9X target proteins (Wang et al., 2014; Yang et al., 2016), which is consistent with our findings. It is, however, important to note that both PR-619 and WP1130 are not specific to the indicated targets. For example, WP1130 is considered a partially selective USP9X inhibitor, as it also targets other DUBs, USP14, USP5, and UCH37 (Kapuria et al., 2010). Similarly, PR-619 is characterized as a broad-spectrum DUB inhibitor (Altun et al., 2011). Therefore, further work is needed to determine whether other molecular players modulated by these compounds might play a role in the observed inhibition of the A/LC-mediated SNAP-25 cleavage. Importantly, there have been efforts to develop more potent and selective inhibitors than PR-619 and WP1130 targeting the same DUBs. For example, recently, a highly selective USP9X inhibitor, FT709, as compared to WP1130, was developed and characterized (Clancy et al., 2021). Future work evaluating more selective DUB inhibitors may pave the road for drug development against BoNT/A intoxication.

Another important finding of this study was identifying Celastrol as a regulator of A/LC ubiquitination and degradation (Figures 5, 6), which demonstrated efficacy against BoNT/A holotoxin mediated SNAP-25 cleavage in both pre-and post-intoxication conditions (Figures 1, 3, 7). Celastrol is a natural product derived from *Tripterygium wilfordii*, with anti-inflammatory and antioxidant activities (Venkatesha et al., 2016). Importantly, celastrol is pharmacologically active, and it can cross the blood-brain barrier, making it the focus of many studies in the context of many human diseases, including central nervous system disorders (Bai et al., 2021). Celastrol has multiple targets in cells (Chen et al., 2018), and its treatment has been shown to lead to degradation of specific proteins via the ubiquitin-proteasome pathway, including FANCD2 and mTOR (Metselaar et al., 2019; Wang et al., 2015; Li et al., 2018), which is in line with our results demonstrating the degradation of BoNT/A LC (Figure 5). Interestingly, Celastrol led to significant degradation of BoNT/A LC; however, although not statistically significant, we have observed an accumulation of ubiquitinated A/LC (Figure 6). It has been demonstrated that Celastrol is also a proteasome inhibitor, and therefore, it may be possible that the degree of A/LC degradation is determined by the balance between celastrol-mediated proteasome inhibition and celastrol-mediated increase in A/LC ubiquitination. A similar mechanism has been previously proposed for celastrol-mediated mTOR ubiquitination and degradation (Li et al., 2018).

Our results showed that b-AP15, NSC632839, MDBN, PYR-41, and SL-01, which do not target USP9X or VCIP135, exhibit significant effects on the degradation of BoNT/A LC at varying degrees (Figure 5). These results may be crucial because other possible mechanisms might be important for the stability of BoNT/A LC, different than VCIP135 and USP9X. Alternatively, these compounds might indirectly affect USP9X or VCIP135, leading to A/LC degradation. Notably, there has been significant research on these compounds, which may help future studies to better understand the roles of the compounds in A/LC degradation. b-AP15 targets DUBs USP14 and UCHL5 in 19S proteasome (Tian et al., 2014; Fukui et al., 2019), resulting in polyubiquitination accumulation (D'Arcy et al., 2011). However, this appears to be context-dependent as it has been shown that b-AP15 treatment can enhance poly-ubiquitination of Smad2 and Smad3 and enhance their degradation in the lysosome (Nan et al., 2016), which is consistent with our findings demonstrating that b-AP15 leads to A/LC degradation and enhances its ubiquitination in a time-dependent manner. NSC-632839 targets DUBs USP2 and USP7 and deSUMOylase SENP2 (Nicholson et al., 2008), but is also reported to induce caspase activation and apoptosis (Aleo et al., 2006). In our analyses, NSC-632839 led to increased degradation of A/LC (Figure 5); however, its effect on A/LC ubiquitination was not significant despite there was a trend of increased A/LC ubiquitination (Figure 6). P22077 is also a DUB inhibitor targeting mainly USP7 and USP47 (Altun et al., 2011). Interestingly, this compound did not exhibit a significant effect on A/LC degradation (Figure 5) and ubiquitination (Figure 6) even though it antagonized BoNT/A holotoxin mediated SNAP-25 cleavage in pre-intoxication conditions, suggesting that the compound might affect other mechanisms, such as entry steps, etc. in the intoxication mechanisms. MDBN is a cell-permeable, irreversible inhibitor of p97/valosin-containing protein that has been shown to promote ubiquitination in cells (Schweitzer et al., 2016), which is in line with our findings. PYR-41 is a cell-permeable E1 ligase inhibitor (Yang et al., 2007); however, it also targets several DUBs and some kinases (Kapuria et al., 2011). NSC-66811 and SL-01 both target E3 ubiquitin-protein ligase Mdm2 (Lu et al., 2006; Li et al., 2011). Taken together, these compounds and their potential targets within the context of BoNT/A LC stability should be further investigated in future studies. Such further characterizations should include analyses to determine whether the lead compounds might directly inhibit the enzymatic activity of A/LC, which is a zinc-dependent endopeptidase (Lebeda et al., 2010). Our analyses demonstrated that the lead compounds did not exhibit a significant effect on cell viability at tested concentrations (1.25, 2.5, 5, 10, and 20 µM), based on the MTT assay (Figure 4). Considering the experimental conditions and the exposure time of compounds in culture, in which they exhibited apparent activity against the toxin, in the initial screen (Figure 1), dose-response (Figure 3), and post-intoxication (Figure 7) analyses, we exposed the cells to compounds for 4 h in our MTT assay. However, future characterizations of the lead compounds should also include further toxicological testings with various compound treatment time points.

An effective strategy to treat botulism patients may involve a combination therapy in which specific immunoglobulins can be used to neutralize the toxin before cellular entry, coupled with drugs that can inhibit the biological activity of the already internalized toxin. Indeed, there are approved anti-BoNT antibody therapies, which are effective before BoNTs gain access into the cells (Rasetti-Escargueil and Popoff, 2019). However, as indicated above, there are currently no approved drugs against the already internalized active toxin (Lin et al., 2019). Only a limited number of BoNT molecules in cells can be sufficient to block neurotransmission (Hanig and Lamanna, 1979), and multiple groups have demonstrated that cleavage of a relatively small fraction of total SNAP-25 by A/LC is sufficient to cause significant inhibition of neuroexocytosis (Keller and Neale, 2001; Meunier et al., 2003; Keller et al., 2004; Beske et al., 2015). Given that A/LC can survive in cells for months (Shoemaker and Oyler, 2013), it would be ideal to eliminate already internalized A/LC to complement approved antibody therapies. Our work in this study focused solely on BoNT/A; however, it is crucial to develop inhibitors that can be effective against multiple BoNT serotypes. It has been shown that BoNT/E is degraded relatively rapidly compared to BoNT/A due to ubiquitination by a different E3 ligase than that of BoNT/A (Tsai et al., 2010). Future work should evaluate whether the ubiquitin-proteasome pathway can also be modulated to target serotype E and potentially other serotypes.

In summary, this study identified a subset of UPP targeting small molecules that inhibits BoNT/A LC activity in cells; two of the most efficacious compounds are DUB inhibitors. This is important as DUBs are considered druggable targets with significant clinical potential for various conditions (Magin et al., 2021). Initial studies described herein provide proof-of-concept data indicating that small molecules targeting UPP can be useful for attenuating BoNT/A intoxication. Modulating BoNT half-life in cells by small molecules can be important for research purposes to understand intoxication/recovery mechanisms and the development of effective countermeasures against botulism.

## Data Availability

The original contributions presented in the study are included in the article/Supplementary Material, further inquiries can be directed to the corresponding author.
